# Towards a better understanding of Rift Valley fever epidemiology in the south-west of the Indian Ocean

**DOI:** 10.1186/1297-9716-44-78

**Published:** 2013-09-09

**Authors:** Thomas Balenghien, Eric Cardinale, Véronique Chevalier, Nohal Elissa, Anna-Bella Failloux, Thiery Nirina Jean Jose Nipomichene, Gaelle Nicolas, Vincent Michel Rakotoharinome, Matthieu Roger, Betty Zumbo

**Affiliations:** 1 CIRAD, UMR Contrôle des maladies, F-34398 Montpellier, France; 2 Centre de recherche et de veille sur les maladies émergentes de l’Océan Indien (CRVOI), 2 Rue Maxime Rivière, 97490 Ste Clotilde, Ile de la Réunion, France; 3 CIRAD-Département “Environnement et Sociétés”, UR AGIRs “Animal et Gestion Intégrée des Risques”, TA C-22/ E, Campus international de Baillarguet, 34398 Montpellier, Cedex 5, France; 4 Institut Pasteur, Unité d’Entomologie médicale, Ambatofotsikely BP 1274, Antananarivo 101, Madagascar; 5 Institut Pasteur, Department of Virology, Arboviruses and Insect Vectors, 25–28 rue du Dr Roux, 75724 Paris, cedex 15, France; 6 Ministère de l’Agriculture de l’Elevage et de la Pêche, Direction de la santé animale et phytosanitaire, Ambatofotsikely, Madagascar; 7 Service de lutte anti-vectorielle, Agence de Santé Océan Indien, Délégation de l’île de Mayotte, BP-410 Mamoudzou, France

## Abstract

Rift Valley fever virus (*Phlebovirus*, Bunyaviridae*)* is an arbovirus causing intermittent epizootics and sporadic epidemics primarily in East Africa. Infection causes severe and often fatal illness in young sheep, goats and cattle. Domestic animals and humans can be contaminated by close contact with infectious tissues or through mosquito infectious bites. Rift Valley fever virus was historically restricted to sub-Saharan countries. The probability of Rift Valley fever emerging in virgin areas is likely to be increasing. Its geographical range has extended over the past years. As a recent example, autochthonous cases of Rift Valley fever were recorded in 2007–2008 in Mayotte in the Indian Ocean. It has been proposed that a single infected animal that enters a naive country is sufficient to initiate a major outbreak before Rift Valley fever virus would ever be detected. Unless vaccines are available and widely used to limit its expansion, Rift Valley fever will continue to be a critical issue for human and animal health in the region of the Indian Ocean.

## Table of contents

1. Disease and transmission

2. Mosquito vectors

3. Virus-vector interactions

4. Diagnosis and surveillance

5. Prevention and control

6. Future of RVF on islands of the Indian Ocean

7. Competing interests

8. Authors’ contributions

9. Acknowledgments

10. References

## 1. Disease and transmission

Rift Valley fever (RVF) is an emerging zoonotic vector-borne disease representing a threat to animal and human health, and livestock production. Abortions and high mortalities in newborns are observed in animals [1,2]. In humans, symptoms vary from a flu-like syndrome to encephalitic, ocular or hemorrhagic syndrome. The case fatality rate of the latter form can be as high as 50% [3].

**Figure 1:**
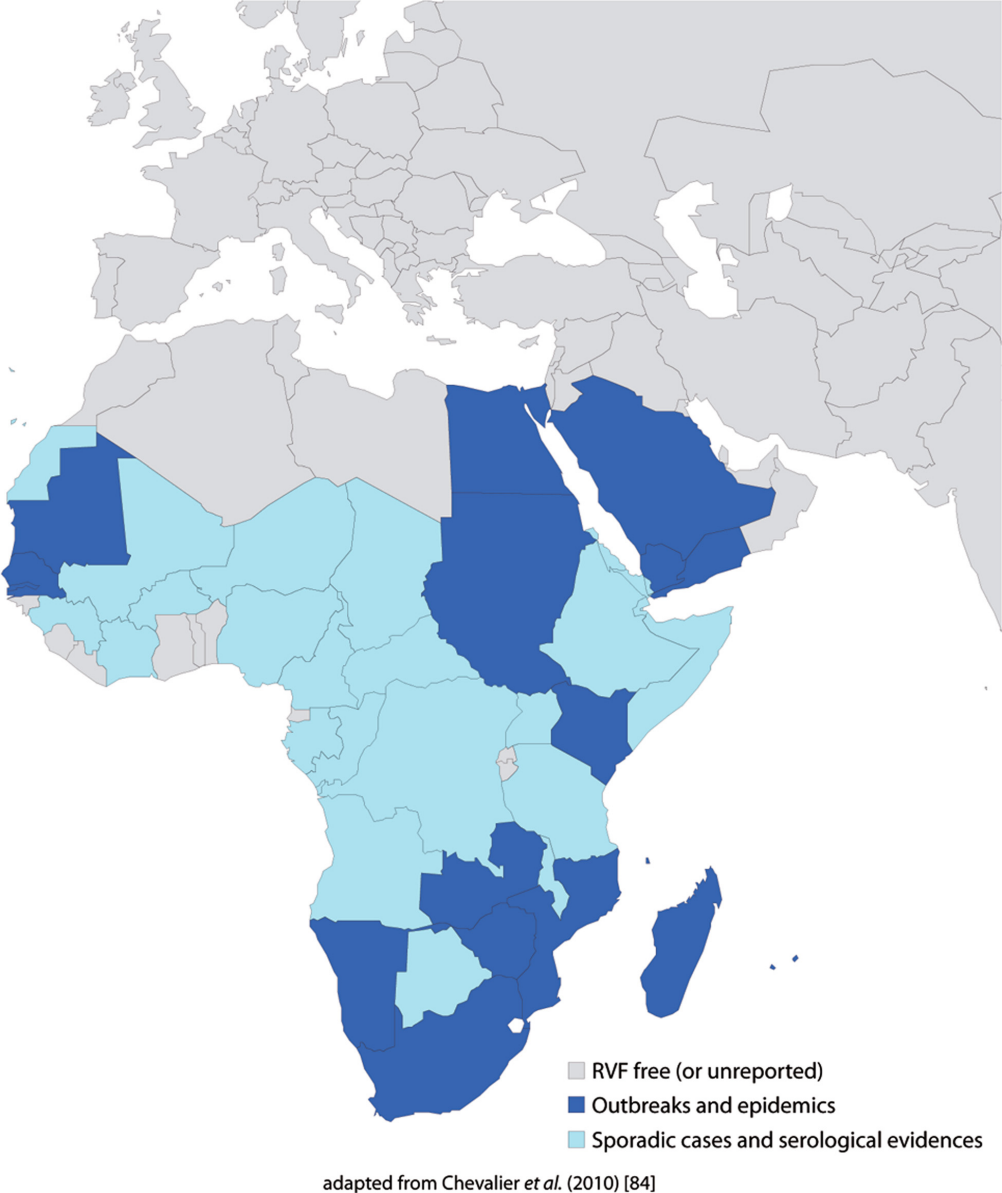
**Geographical distribution of Rift Valley fever.** Rift valley fever is historically endemic to many countries of sub-Saharan Africa. It spread from continental Africa to Madagascar in 1991 and in the Arabian Peninsula in 2000. In Mayotte in the Indian Ocean, RVF cases have been reported since 2004.

Since its first isolation in 1930 in Kenya [4], RVF outbreaks have been reported in most sub-Saharan countries, especially the Rift Valley in Kenya and Tanzania [5] (Figure 1). Subsequent outbreaks with human cases have been reported in South Africa [6] and the Nile Valley from Sudan to the Egyptian delta [7]. The disease spread from continental Africa to Madagascar in 1991 [8-11] and in the Arabian Peninsula in 2000 [12]. In Madagascar, RVFV was isolated for the first time in 1979 from pools of mosquitoes captured during the rainy season in the primary rain forest of Perinet, Moramanga district [13]. The most recent RVF outbreaks were detected in Somalia (2006–2007) [14], Kenya (2006–2007) [15], Tanzania (2007) [14], Sudan (2007–2008) [16], Madagascar (2008–2009) [17], South Africa (2008, 2009, and 2010) [18], Mauritania (2010) [19], Botswana (2010) [20], and Namibia (2010) [21]. In Mayotte, sporadic cases in livestock have been recorded since 2004 with human cases detected in 2007–2008 and 2011. While RVF was originally associated with livestock mortality, recent outbreaks have resulted in increased fatality rates in humans [16].

**Figure 2:**
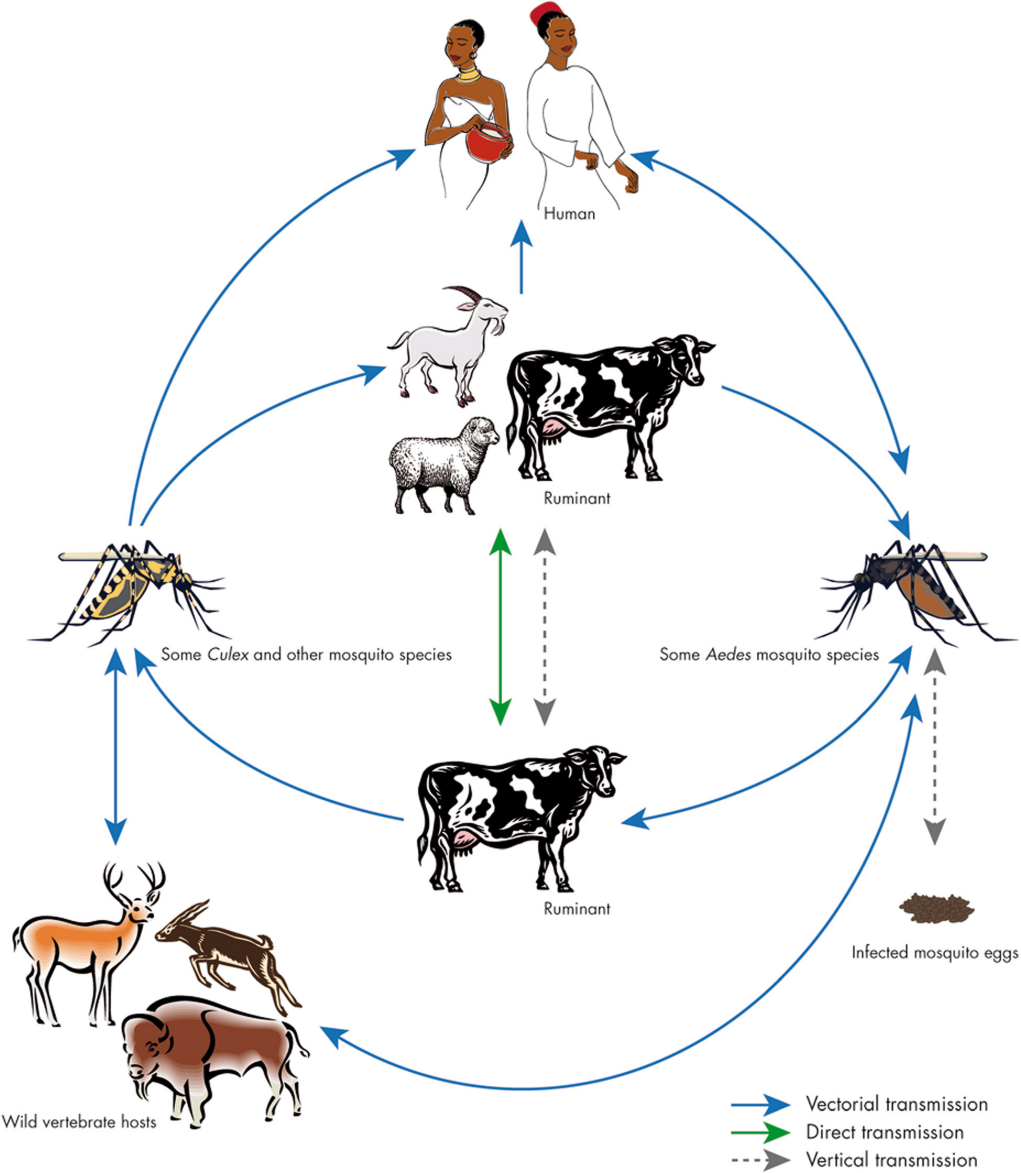
**Cycle of Rift Valley fever.** The virus can be maintained in an enzootic cycle involving *Aedes* mosquitoes which are able to transmit the virus vertically to their offspring. Epizootic outbreaks are often linked with unusual rains or warm seasons, favoring the hatching of infected *Aedes* eggs that are then able to initiate the virus circulation. Subsequently, large numbers of secondary vectors belonging to the *Culex* genus could be infected and induce the emergence of epidemic/epizootic outbreaks. Transmission to humans occurs through direct contact with high virus loads when aborted fetuses are manipulated.

Rift Valley fever virus (RVFV) is transmitted among ruminants by mosquito bites mainly belonging to the *Aedes* and *Culex* genera and by direct contact with body fluids of viremic animals. Moreover, biological or mechanical transmission of RVFV was reproduced experimentally with other hematophagous flies but field relevance of these transmission routes are still unclear [22,23]. Humans are mainly infected by close contact with blood, excreta of infected animals, consumption of raw milk [24], and in some rare cases, through mosquito bites [25] (Figure 2).

RVFV circulates between animals within an enzootic cycle during most years, but may become epizootic during wet years in regions such as East Africa. Virus is maintained during dry seasons in desiccation-resistant eggs of several *Aedes* species which have acquired the virus by vertical transmission [26]. For example, in East Africa, flooding of natural excavations lead to the hatching of large numbers of *Aedes* (*Aedimorphus* and *Neomelaniconion* subgenera) eggs initiating viral circulation. Movements of viremic animals along trade routes have been suspected to be responsible for the virus spreading [27]. Unless vaccines are used on a large scale in Africa, RVF will continue to be a significant problem with the fear of being introduced into western countries.

In the Indian Ocean, RVF was described in Madagascar, Mayotte and other islands of Comoros. In Madagascar, RVFV was first isolated in 1979 from mosquitoes [28]. The isolated viral strains were closely related to Egyptian strains. Later, epizootics in 1990 involved viral strains genetically close to strains from Zimbabwe [11,29]. The first human cases were only reported during the 2008 outbreak; 417 cases were suspected with 59 laboratory confirmed, and 19 deaths [17]. The proximity with East Africa may favor recurrent RVF introduction via livestock trade [29]. A survey conducted in 2009 in cattle from a pilot area in Madagascar highlands demonstrated a recurrent circulation of RVFV [30]. In this temperate and mountainous region, the climate is not favourable to RVFV vectors. Therefore, the involvement of cattle in virus circulation and persistence was suspected. In the same area, two distinct trading practices have been described [31]: usual trade and a traditional barter practice named *kapsile*. To conclude the barter, the applicant has to exhibit his cattle allowing frequent contact between animals and people. Social network analysis methods suggested that networks could be formed by preferential attachment mechanisms, due to a better reputation of some breeders or villages. The results highlighted the need for a careful description of exchange practices for the understanding of the RVFV circulation mechanisms. Due to links with markets located in RVFV-affected areas during the 2008–2009 outbreak, the usual trade network could support virus introduction from other parts of Madagascar. A protective effect of the village distance to the nearest water point which suggested a vector-borne transmission could partly support disease transmission in the highlands area.

In Mayotte, a human case was reported in August 2007 suggesting an autochthonous circulation of RVF on this island of the Comoros archipelago [32]. Amongst the 488 sera sampled from ruminants, 32.8% were found to be serologically positive during the 2009 dry season [33]. Serological surveys of ruminants showed a RVF circulation on Mayotte as early as 2004 without any human or animal cases detected [34].

## 2. Mosquito vectors

Eighteen out of more than 65 mosquito species described as potential vectors of RVFV worldwide, have been found in Madagascar [28]. *Aedes* species of the subgenus *Neomelaniconion*, incriminated in the vertical transmission of RVFV in Kenya were also present on the island [28]. Four periods can be defined in RVF history in Madagascar:

(i) 1979 with the first detection of RVFV. The virus was first isolated in 1979 from multispecific pools of *Anopheles* (*An. coustani, An. fuscicolor, An. pauliani, An. squamosus), Culex* (*Culex simpsoni, Cx. vansomereni, Cx. antennatus, Cx. quinquefasciatus, Cx. annulioris, Cx. univittatus), Mansonia uniformis* and *Coquillettidia grandidieri*[35] during the rainy season in the forest area of Périnet in the district of Moramanga*.* However, this study was not able to give the accurate implication of each species in the RVF transmission or their role in the maintenance of the virus during the inter-epizootic period.

(ii) 1990–1991 with the first epizootics. During the first epizootics of RVF in the Malagasy livestock described in the east coast and the highlands, respectively, in 1990 and 1991, thousands of mosquitoes were collected but no virus was isolated [8-11].

(iii) 1993–2008 with RVFV detected in Malagasy cattle during the inter-epizootic period. From 1993 to 2008, the virus was not detected in more than 150 000 mosquitoes tested (*Anopheles, Culex, Aedes, Mansonia, Aedemomyia, Coquillettidia, Eretmapodites, Mansonia,* and *Uranotaenia*) despite its circulation in livestock and its maintenance at a low level [28,36,37].

(iv) 2008–2009 with the last epizootic. Recently, in 2008–2009, RVF was recorded in humans and cattle in several districts of Madagascar [17] with a broad distribution of the virus on the island [38]. These re-emergences during two rainy seasons suggested the role of mosquitoes in the transmission of RVF: (1) RVFV has been detected and isolated from three mosquito species, namely: *An. coustani, An. squamosus/cydipis* and *Cx. antennatus*, captured in three districts of the province of Fianarantsoa, in the Central Highlands [39]; (2) a risk factor study carried out with cattle in 2009 reinforced this assumption [30]; and lastly, (3) their zoophilic and opportunistic anthropophilic behavior in the absence of household hosts allow assuming the implication of these species in the RVF transmission in Madagascar [40]. Other RVF vector species identified as a potential vector in Saudi Arabia (*Cx. tritaeniorhynchus* in 2000 [41], Egypt (*Cx. pipiens* in 1977–78 [27,42], Kenya (*Ae. durbanensis* and *An. pharoensis* in 1981–84 [26]) have also been found in Madagascar. During these episodes of RVF circulation in Madagascar, no studies on vector competence were undertaken. Indeed, the implication of none of these eighteen species involved in the transmission of RVFV in Madagascar has been corroborated. But according to their abundance, their trophic preference towards animals (especially, zebu) and their involvement in the transmission of RVFV in other countries, *An.* coustani, *An squamosus/cydippis, Cx. antennatus, Cx. univittatus, Cx. tritaeniorhynchus, An pharoensis, Cx. pipiens, Cx. quinquefasciatus, Ae. durbanensis and Ae*. (subgenus) *Skusea* could act as potential vectors in Madagascar*.* The last two species are abundant only in the southern part of the island [43].

On the Comoros Islands, RVFV was isolated for the first time in 2007 consecutively to important epizootics occurring in East Africa in 2006 [32]. In 2008–2009, the presence of RVFV was confirmed in domestic ruminants [33,34] and in some inhabitants of Mayotte [44]. The region of the Indian Ocean presents a particular interest for RVF emergence since human displacements and animal exchanges with continental Africa are important. In addition, island ecosystems may present a particular biodiversity (usually a low specific richness but a high level of endemism due to adaptive radiation) favouring the occurrence of particular virus/vector/vertebrate systems. Indeed, a recent inventory in Mayotte described 40 mosquito species belonging to 9 genera.

In Mayotte, an ongoing 2-year survey has been carried out to describe diversity, population dynamics and host-feeding patterns of mosquitoes in five ruminant farms. Adult collections were performed every 4 weeks during 3 consecutive nights using light and CO_2_-baited traps. Moreover, twenty-four-hour collections were made on one site using human-, sheep-, and goat-baited traps to describe host-feeding patterns and circadian activities. After a one-year survey, about 7900 mosquitoes belonging to 19 species and 4 genera (*Culex*, *Eretmapodites*, *Aedes*, *Anopheles*) were collected during 24–48 sessions of adult collections per site and 6 sessions of host-baited trap collections. *Culex quinquefasciatus* and *Eretmapodites quinquevittatus* were the most abundant species collected on humans and sheep. *Cx. quinquefasciatus* and *Er. quinquevittatus* were shown to develop low to moderate infection rates [45]. In host-baited traps, two anthropophilic mosquitoes *Culex antennatus* and *Aedes albopictus,* and *Culex decens*, a non-mammalophilic mosquito, were found abundantly. Other species were captured at lower densities in CO_2_ or light-baited traps. *Eretmapodites subsimplicipes* and *Aedes aegypti* were found in host-baited traps but not in CO_2_ or light-baited traps highlighting the need for complementary methods to be included such as larval collections to better describe mosquito diversity and abundance.

## 3. Virus-vector interactions

Three major RVFV lineages are described corresponding to Egyptian, West African and East African strains [46]. Interestingly, the virus isolated in Madagascar in 1990 belongs to the same lineage as the Kenyan isolate of 1997 [47]. RVFV strains isolated from the Arabian Peninsula were closely related to the Kenyan isolates [41,48,49]. By analyzing more carefully each of the three segments of the genome, several strains resulted from reassortments between strains from different genotypes [50]. Such a phenomenon is not unexpected for a virus with a segmented genome but this could be a strategy for selecting more virulent viral strains.

Most arboviruses are RNA viruses, although they use a variety of strategies to ensure their replication and transmission. The feature that best distinguishes RNA is their high mutation rate during replication ranging from 10^-3^-10^-5^ substitutions per nucleotide and per round of copying [51]. The main factor contributing to such high mutation rates is a lack of proof-reading repair activities that is associated with RNA replicases [52]. High mutation rates, short replication times and large population sizes, ensure the existence of the quasispecies genome pool [53]. However, arboviruses are relatively stable in nature suggesting that the alternating host cycle (between vertebrate and invertebrate hosts) constrains viral evolution by a strong conservative sequence selection. Serial passages in a single cell type (mammalian or insect cells) led to the loss of RVF virulence. Large deletions were observed in the NSs gene. Thus, genetic material non-essential to viral replication such as NSs is rapidly eliminated leading to the loss of virulence [54]. It is tempting to speculate that direct transmission among animals or from animals to humans may favor the attenuation of NSs leading to the maintenance of avirulent RVFV strains. Virulence is likely to be restored when alternation is initiated again. Indeed, vertebrates are subject to acute infections with clearance of the virus triggered by immune defense when vectors sustain persistent viral replication becoming the site of genetic changes such as reassortments. Such rearrangements may lead to restore virulence with the acquisition of a complete NSs gene in the course of virus replication [50]. This result agrees with observations on the natural evolution of RVF outbreaks whose intensity declines with increasing herd immunity and declining vector populations.

## 4. Diagnosis and surveillance

As described in many African countries, the virus may circulate at a very low level, silently, without or with few clinical signs: this cryptic transmission is extremely difficult to detect [55]. In Mayotte, in 2004, where a seroprevalence of up to 22% (130 animals tested) was estimated in small ruminants without any clinical symptoms, RVF was diagnosed in a human case presenting brain disorders. Susceptible animals were present in densities high enough to ensure virus circulation but too low to induce waves of abortions and animal mortalities.

To detect RVFV, several approaches are available: molecular detection (RT-PCR, real-time quantitative RT-PCR), virus antigen detection, and anti-RVF IgM or IgG antibody detection [56]. The main drawback of medical laboratories in the Indian Ocean region remains the weakness of diagnostic tools. Indeed, some diagnosis of RVF can only be achieved in well-furnished laboratories, with a range of standardized diagnostic reagents involving experienced personal used to manipulating serial numbers of strains. Another issue is the difficulty in maintaining the cold chain, particularly when distances are important as in Madagascar, between the field and the laboratory; samples must be stored at −80 °C for further viral isolation. Thus, appropriate containers and ice packs for transportation allowing keeping serum samples between −20 °C and +4 °C, should be provided to veterinary services in charge of sample collections. This highlights the need of an early warning system to improve transfer between the field and the laboratory.

Depending on the epidemiological status of the country, surveillance has its main purpose. In La Reunion, Maurice and Seychelles which are RVF-free islands but at risk for introduction, early detection of the disease is a priority. In Mayotte, Comoros and Madagascar where RVF is probably endemic, the priority is the detection of any increased incidences preceding outbreaks. The epidemiological surveillance was heterogeneous between the different islands: some countries had their own network such as Madagascar, Mayotte or La Reunion Island and some others received direct epidemiological information from the field (Comoros, Maurice, and Seychelles). Since then, a regional epidemio-surveillance network called “AnimalRisk – OI” has been set up. All stakeholders in the Indian Ocean involved in animal health are included in this network (veterinary public health and research institutes). A steering committee composed by the chief veterinary officers or their deputy is in charge of defining the diseases under surveillance, RVF mainly, and data management. Once a month, the steering committee disseminates collected epidemiological data through web conferences and later, a quarterly epidemiological report is produced. In endemic countries, sentinel herds have been followed up for one year and half are used to assess the seroconversion rate, to detect any clinical cases that could be attributed to RVF and finally, to confirm the circulation of RVFV despite very few notified symptoms [57]. Once the disease is detected in humans, it is usually well-established in animal populations. Nevertheless in Madagascar and Mayotte in 2008, livestock infections were detected after severe human cases were reported [32,58], highlighting the lack of collaboration between human and animal health services.

Given the frequent low specificity of clinical signs, syndromic surveillance may be a useful and cost-effective tool to help in controlling RVF. Indeed, this methodology allows minimizing the main limitations of the passive surveillance systems: (i) reducing the time lag between the onset of the outbreak and the diagnosis, (ii) using a nonspecific case definition to increase the sensitivity, and (iii) minimizing the under-reporting by the systematic and continuous screening of information at an earlier stage of the disease process. On the human side in Mayotte in 2009, the surveillance of acute febrile syndromes in humans allowed to detect 10 human RVF cases [59].

The development of models based on climatic indicators and vegetation index remains promising for risk-based surveillance implementation. These models are available for Eastern Africa and allow forecasting RVF outbreaks many weeks before. However, their predictive performance was shown to be low for Madagascar and South Africa, and needs to be improved and adapted to specific Malagasy ecological and climatic conditions [60,61].

## 5. Prevention and control

Several control measures are described usually including the following: (i) control of livestock movements with respect to trade and export; (ii) vector control with an emphasis on larvicides in vector breeding sites rather than aerial sprayings targeting adults or (iii) vaccination of livestock.

The role of livestock movements in RVF spread at short or long distances -trade, transhumance- has already been shown [31,48,62]. As a matter of fact, a large livestock trade exists or existed between countries in the Indian Ocean and countries of Eastern Africa where the disease is endemic, and phylogenetic studies strongly suggest that RVFV has been introduced in the Indian Ocean by ruminant trade [29,63].

Because RVF could be introduced through the importation of domestic ruminants from infected countries, although this could only occur if importation took place within the short incubation period for the disease, adoption of the recommended guidelines of the OIE International Animal Health Code for such importations would prevent this risk. When importing from infected countries, veterinarian authorities should require for domestic ruminants the presentation of a veterinary certificate attesting the following:

1. Vaccinated animals (a) showed no clinical signs of RVF on the day of shipment; (b) were vaccinated using a vaccine complying with the standards described in the OIE Manual not less than 21 days and not more than 90 days prior to shipment; (c) were kept in a quarantine station in the country of origin for the 30 days prior to shipment and showed no clinical sign of RVF during that period.

2. Unvaccinated animals (a) showed no clinical sign of RVF on the day of shipment; (b) were subjected to the diagnostic tests for RVF with negative results within 30 days before entry into quarantine; (c) were kept in a quarantine station in the country of origin for the 30 days prior to shipment and showed no clinical sign of RVF during that period; (d) were subjected to the diagnostic tests for RVF with negative results not less than 14 days after entry into quarantine; (e) were protected from insect vectors during quarantine and transportation to the place of shipment.

But to date, all these recommendations are often disregarded between Tanzania and Comoros. Indeed, since 2002, importation of live animals in Comoros from Tanzania has been common, increasing the risk of introducing continental pathogens or vectors as illustrated with outbreaks of East Coast fever in 2003 and 2004 in Grande Comore [62]. Once RVFV is introduced, it is quite difficult to prevent its spread (i) between the neighboring islands because of no quarantine park and illegal trade and (ii) throughout countries, such as Madagascar, where the large-scale movement of cattle is common and often uncontrolled.

The timing of the events associated with the 2007–2008 outbreak in the Indian Ocean and molecular epidemiological studies also support RVFV importation from East Africa, possibly even during the 2006–2007 outbreak. Although most cases in the Indian Ocean (Comoros, Mayotte and Madagascar) were reported from January through April 2008 [32,64], epidemiologic evidence has linked the 2008–2009 epizootics to that occurring on the mainland a few years earlier. Especially, clinical signs of RVF (human or animal cases) were observed in early 2007 on these islands, and retrospective investigations revealed that RVFV had been circulating in livestock at least since December 2007 [38]. Maurice must also be considered as an island at-risk because of regular importations of goats from Kenya or cows from South Africa; even if there are severe quarantine measures at Richelieu station, such as surveillance of any clinical signs, random serological sampling, and quarantine duration from 15 days to 2 months, an emphasis should be made on surveillance to detect any sign of RVF disease.

Beside the trade control, a safe vaccine is now available for livestock [65-67], which is probably an efficient way to protect both animals and humans interrupting the virus transmission in endemic areas; even if it could be responsible for the generation of recombinant viruses when used in ongoing infection areas [68]. However, socio-economic studies are needed to assess the sustainability and the acceptability of measures by breeders in the Indian Ocean context. Other ways to control the spread of RVF involve control of the vector and protection against their bites. Larvicide controls of mosquito breeding sites are the most effective measure of vector control but are applicable only if breeding sites can be clearly identified and are limited in size and extent. During periods of flooding, however, the number and extent of breeding sites are usually too extended for insecticide treatments. Besides the financial cost [69], ecological and health issues associated with the extensive use of insecticides should be considered. Lastly, since the major route of human infection is direct exposure to infected animals [69], information of people may also ensure appropriate slaughtering and consumption practices, thus decreasing the risk of infection to humans.

## 6. Future of RVF on islands of the Indian Ocean

Following the 2007 Eastern Africa outbreak and the detection of indigenous human cases in Mayotte in 2008 [32], a global qualitative assessment of the risk of outbreak for Mayotte and La Reunion Island, was performed by the French Agency for Food Safety (AFSSA, now ANSES) [70]. The conclusions of this assessment were that the following: (i) the risk of outbreak in these two islands would be rather due to the introduction of a viremic animal coming either from Madagascar, Comoros or Eastern African coasts; (ii) this risk would be rather reduced for La Reunion Island located far from endemic areas and where controls of imports are drastic; (iii) in Mayotte, illegal introductions of ruminants are frequent enough to justify more controls of livestock imports; (iv) in Mayotte again, due to low animal densities, the risk for RVF to become endemic was estimated to be very low. Therefore, a systematic vaccination of livestock was not recommended [71]. Regarding the risk of human infection, AFSSA recommended developing and/or reinforcing the information of potentially exposed people such as breeders, veterinarians, slaughterhouse workers, taking into account cultural habits. For surveillance, the implementation of an active monitoring of sentinel herds in the main entry sites of imported ruminants appeared as a priority.

The future of RVF in the islands of the Indian Ocean remains unpredictable. Their proximity with the Eastern African coast makes these islands permanently at risk. Eco-climatic, economical, geographical and cultural contexts are so diverse that each island must be considered as a special case. La Reunion Island, which is geographically isolated, is rather protected whereas the Comoro Islands appear to be highly exposed. Seychelles and Maurice remain RVF-free but animal trades with continental Africa keep them vulnerable. The way the virus spread in 2008 in Madagascar demonstrated how this country is susceptible to this disease. However the mechanisms involved in the virus spread and its persistence on the island remain partially unknown. From a global point of view, scientific knowledge on RVF epidemiology in this region is poor and needs to be improved. The existing predictive models are not relevant, justifying research investment to identify the main emergence and spread determinants, quantify the transmission parameters, and build models that should be used (i) to predict the risk of emergence in space and time, and (ii) to evaluate the efficiency of surveillance and control measures in each context.

## 7. Competing interests

The authors declare that they have no competing interests.

## 8. Authors’ contributions

TB, EC, VC, NE and ABF drafted the manuscript. TNJJN, GN, VMR, MR and BZ were involved in revising the manuscript. All authors have read and approved the manuscript.
